# Editorial: Fungal pathogens causing the grapevine trunk diseases- biology and identification

**DOI:** 10.3389/ffunb.2023.1186166

**Published:** 2023-04-12

**Authors:** Gonzalo A. Díaz, Pierluigi Reveglia, Liliana Lucia Tomoiagă, Veronica Sanda Chedea

**Affiliations:** ^1^ Laboratorio de Patología Frutal, Departamento de Producción Agrícola, Facultad de Ciencias Agrarias, Universidad de Talca, Talca, Chile; ^2^ Research Institute for Sustainable Agriculture, Spanish National Research Council (CSIC), Córdoba, Spain; ^3^ Research Department, Research Station for Viticulture and Enology Blaj (SCDVV Blaj), Blaj, Romania

**Keywords:** fungal pathogens, *Vitis vinifera*, grapevine dieback, grapevine nursery propagation material, phenolic compounds

Grapevine (*Vitis vinifera* L.) is widely cultivated as an important fruit crop destinated for wines, table grapes, and raisins. However, grapevines are plagued by a variety of diseases each year, depending on the causal agents, cultivar susceptibility, and climate location, and these diseases are limiting factors in commercial vineyards and nurseries (Wilcox et al., 2015; Muntean et al., 2022). In this sense, Grapevine Trunk Diseases (GTDs) are one the most critical factors affecting vineyards’ productivity and quality (Wilcox et al., 2015; Kaplan et al., 2016; Gramaje et al., 2018; Gispert et al., 2020; Azevedo-Nogueira et al., 2022; Kenfaoui et al., 2022). GTDs are diseases caused by phytopathogenic Ascomycetes (*Cadophora* spp., *Phaeomoniella chlamydospora*, *Phaeoacremonium* spp., Diaporthaceae, Nectriaceae, Diatrypaceae, and Botryosphaeriaceae species, among others) and Basidiomycetes fungi species into genera *Arambarria, Fomitiporia, Fomitiporella, Inocutis* and *Inonotus* (Mugnai et al., 1999; Díaz and Latorre, 2014; Cloete et al., 2015; Pildain et al., 2017; Gramaje et al., 2018; Azevedo-Nogueira et al., 2022; Kenfaoui et al., 2022). These pathogens invade the lignified tissues, causing necrosis and producing hard and soft decay in the grapevine wood. They can affect both grapevines established in the field and propagation material in nurseries (Mugnai et al., 1999). In mature vineyards, Esca complex dieback, Eutypa dieback, Botryosphaeria dieback, and Phomopsis dieback are the main GTDs, while in young vineyards, the major GTDs are Petri disease and Black-foot disease (Gramaje et al., 2018; Azevedo-Nogueira et al., 2022; Kenfaoui et al., 2022).

The present Research Topic was launched in late 2021 in the section ‘Fungi-Plant Interactions’ of Frontiers in Fungal Biology, including four original research (Carbone et al.; Wallis et al.; Travadon et al.; Díaz and Latorre) by 15 authors.

One of the most prominent sources of spreading GTD pathogens is through infected propagation material (Gramaje et al., 2018; Claverie et al., 2020). Nevertheless, there is a lack of information about the occurrence of GTD pathogens during the propagation process and the health status of the plants in local nurseries. Considering this background, Carbone et al. in their article ‘*Grapevine nursery propagation material as a source of fungal trunk disease pathogens in Uruguay* ‘ identified the GTDs and associated pathogens affecting nursery grapevine plants produced in Uruguay and quantified their incidence within two years: 2018 and 2019. Moreover, the authors wanted to investigate the steps by which the incidence of GTDs increases during the local nursery propagation process.

Their results showed a high incidence of GTDs in nursery grapevines produced in Uruguay, regardless of the type of propagation material. GTDs’ wood symptoms were observed in all propagation stages. Over 80% of the finished nursery vines were affected by at least one GTD pathogen in both years. After phylogenetic analysis, the 180 selected isolates were placed into eight genera within 22 fungal species associated with GTDs. *Phaeoacremonium oleae* and *Diaporthe terebinthifolii* have been isolated for the first time from grapevine worldwide. In conclusion, the results of Carbone et al. provide a valuable frame of the current health status of nursery vines produced in Uruguay, suggesting that a sanitation program is required to reduce the incidence of GTDs.

In the study performed by Wallis et al. titled ‘*Mixed infections of fungal trunk pathogens and induced systemic phenolic compound production in grapevines*’, they compared the effect of sequential co-inoculations with *Diplodia seriata*, *Neofusicoccum parvum*, or *Phaeomoniella chlamydospora* on changes in phenolic compounds and lengths of lesions in young grapevines. The results showed that the effects of fungal infections on phenolic compounds were variable for *N. parvum* and *P. chlamydospora*; however, *D. seriata* was associated with significantly higher concentrations of phenolic compounds distally. These results demonstrate that the effects of one fungal trunk pathogen infection are generally unable to distally affect another long-term, albeit shifts in host phenolics and other plant defenses do occur.

Identifying fungal trunk pathogens is very important for the knowledge of the biology and epidemiology of GTDs. The identification of the causal fungi is critical to implementing appropriate management strategies. Etiology of the GTDs from grapevines cultivated in Washington (wine grapes) and California (table grapes) was studied by Travadon et al., and the work was titled ‘*Fungal species associated with grapevine trunk diseases in Washington wine grapes and California table grapes, with novelties in the genera Cadophora, Cytospora, and Sporocadus*’. The authors aimed to identify 36 species from 112 isolates, with a combination of species that are new to science, are known causal fungi of grapevine trunk diseases, or are known causal fungi of diseases of other woody plants. The novel species *Cadophora columbiana*, *Cytospora macropycnidia*, *Cytospora yakimana*, and *Sporocadus incarnatus* were formally described as being new to science. Other six species were also identified as *Cytospora viticola, Diatrype stigma, Diplodia seriata, Kalmusia variispora, Phaeoacremonium minimum*, and *Phaeomoniella chlamydospora*. Dominating the fungal community in Washington wine grape vineyards were species in the fungal families Diatrypaceae, Cytosporaceae, and Sporocadaceae, whereas in California table grape vineyards, the dominant species were in the families Diatrypaceae, Togniniaceae, Phaeomoniellaceae, and Hymenochaetaceae.

*Phaeomoniella chlamydospora* is one of the most prominent trunk pathogens associated with Petri and Esca-like diseases (Mugnai et al., 1999; Díaz and Latorre, 2014). Several investigations have highlighted that one of the main sources of natural infections is the air-borne conidia dispersed onto fresh pruning wounds from pycnidia. Thus, investigating the duration of *P. chlamydospora* in pruning wounds is fundamental to developing an effective management strategy for Petri and Esca diseases. *P. chlamydospora* is one of the main GTDs pathogens in Chile. Nevertheless, the duration of the susceptibility of grapevine pruning wounds for this pathogen is still unknown.

In this framework, Díaz and Latorre in their article ‘*Duration of the susceptibility of pruning wounds of different ages to infections by Phaeomoniella chlamydospora on grapevine* cv. *Cabernet Sauvignon in Central Chile*’ evaluated the period of susceptibility of pruning wounds artificially inoculated by *P. chlamydospora* in different periods ranging from 1 to 45 days. They evaluated rooted cuttings and spurs from two consecutive seasons in two Cabernet Sauvignon vineyards in Central Chile. Their results showed that pruning wounds remained susceptible to *P. chlamydospora* for up to 45 days after pruning. These data agreed with those previously reported from other vineyards worldwide. The results highlight that a single fungicide application could not be sufficient to avoid the infection, and further studies evaluating the proper number of applications of fungicides and biocontrol agents are needed.

## Author contributions

All authors listed have made a substantial, direct, and intellectual contribution to the work and approved it for publication.
